# Pulmonary Empty Spaces: Silicone Embolism—A Decade of Increased Incidence and Its Histological Diagnosis

**DOI:** 10.1155/2016/3741291

**Published:** 2016-01-27

**Authors:** Kirill Lyapichev, Felix Manuel Chinea, Julio Poveda, Jeniffer Pereda, Pablo A. Bejarano, Monica T. Garcia-Buitrago

**Affiliations:** ^1^Pathology and Laboratory Medicine Department, Miller School of Medicine, University of Miami, Miami, FL 33136, USA; ^2^Department of Radiation Oncology, Miller School of Medicine, University of Miami, Miami, FL, USA; ^3^Kornberg School of Dentistry, Temple University, Philadelphia, PA, USA; ^4^Department of Pathology, Cleveland Clinic Florida, Weston, FL, USA

## Abstract

Pulmonary embolism (PE) is a critical complication related to multiple disorders and different medical or cosmetic procedures. This case report presents two patients who were admitted for respiratory symptoms in the setting of previously receiving silicone injections for cosmetic purposes and were diagnosed with silicone pulmonary embolism. The relevance of including questions about all cosmetic procedures as a part of a medical history is highlighted, in particular about silicone injections. The diagnosis is confirmed by histological means. Additionally, our review showed the change of most common sites of silicone injections and a significant increase in cosmetic procedures causing silicone embolism during the past twelve years.

## 1. Introduction

Pulmonary embolism (PE) is a critical complication related to various disorders such as deep vein thrombosis (DVT), cardiac arrhythmia, certain coagulopathies, and bacterial endocarditis, trauma. Each year, more than 0.6 million people in the United States suffer from pulmonary embolism with about 50,000 resulting in death [1]. An additional risk is observed in patients with a history of medical or cosmetic procedures that may provide an etiology for patients presenting with respiratory symptoms, especially if they were performed by unlicensed individuals. We present two unusual cases of respiratory syndrome, an analysis of the published literature on silicone embolism from 2004 to 2015, and a comparison with a previous review spanning 40 years, from 1965 to 2004.

## 2. Case Presentations

### 2.1. Clinical Case #1

A 24-year-old African American female presented at four-week gestation with a nonproductive cough and severe shortness of breath that began four days prior with progressive worsening. The patient reported no history of recent travel. Oxygen was administered via nasal cannula and was well tolerated.

On physical examination, the patient was tachycardic (126 bpm) and tachypneic (36 breaths/min); temperature was 36.9°C; blood pressure was 132/81 mm Hg; and peripheral oxygen saturation was 98%. Pulmonary auscultation revealed diffuse wheezing and diminished breathing sounds in bilateral lung bases.

**Figure 1:**
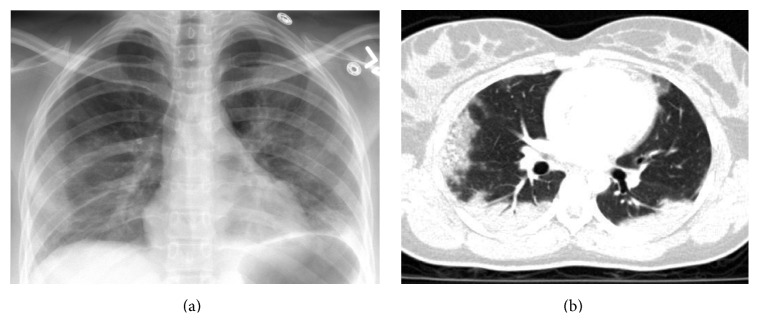
(a) Portable chest X-ray showing bilateral lung opacities with relative sparing of the right upper lobe and left pleural effusions. (b) CT scan of chest showing multifocal and bilateral, predominantly peripheral airspace opacities.

Echocardiogram ruled out valvular disease and myocardial wall abnormalities. Laboratory tests at the time of admission revealed an arterial blood gas analysis within normal limits, anemia (hemoglobin 11.3 mg/dL, hematocrit 34.1), and a normal platelet count (339,000). Chest X-ray revealed bilateral lung opacities with relative sparing of the right upper lobe and left pleural effusions (Figure 1(a)).

Computed tomography (CT) scan showed multifocal, bilateral, predominantly peripheral airspace opacities (Figure 1(b)). A nonspecific differential for this pattern of distribution included eosinophilic lung disease, sarcoidosis, pulmonary infarction, cryptogenic organizing pneumonia, and chronic interstitial lung disease with atelectatic components at the dependent regions of both lungs. No pulmonary emboli to the segmental arteries were found.

She was admitted for treatment of community-acquired pneumonia and developed hemoptysis two days after admission, only completing four days of azithromycin and ceftriaxone. A bronchoscopy with lavage was performed. The bronchial cytology sample showed numerous macrophages, some with cytoplasmic vacuoles, and abundant blood, suggestive of diffuse alveolar hemorrhage.

She was subsequently transferred to the Medical Intensive Care Unit (MICU) with a differential diagnosis of systemic lupus erythematosus, Wegener's granulomatosis, pulmonary capillary hemangiomatosis, and other autoimmune and infectious diseases. Autoimmune laboratory workup for anti-beta 2 glycoprotein, antistreptolysin, ANA, anti-dsDNA, anticardiolipin, anti-GMB, anti-smooth muscle Ab, Anti-Jo, ssA, ssB, RF, p-ANCA, and c-ANCA showed negative results, ruling out the aforementioned autoimmune etiologies.

The patient tested negative for HIV. An infectious disease panel performed on the bronchoalveolar lavage samples of right and left lower lobes testing for HSV, CMV, EBV, PCP, atypical pneumonia panel (*M. pneumoniae*,* C. pneumoniae*, and* L. pneumophila*), viral panel (metapneumovirus, rhinovirus, influenza viruses A and B, RSV A and RSV B, parainfluenza viruses 1, 2, and 3, and adenovirus),* Aspergillus* galactomannan, and* Legionella pneumophila* showed negative results. Thus it decreased the likelihood of any previously mentioned infectious diagnosis.

**Figure 2:**
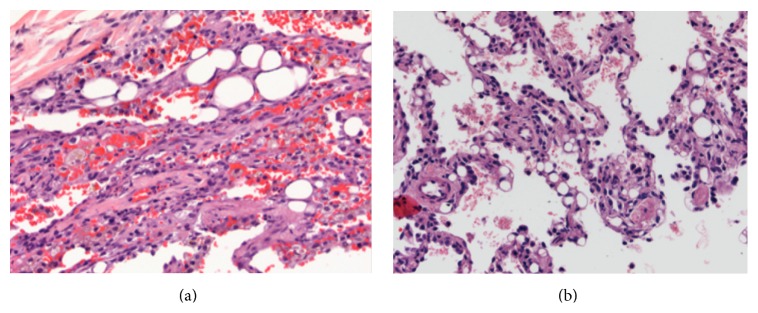
(a) Lung parenchyma with intra-alveolar hemorrhage, multiple vacuoles, macrophages, and dilated capillaries (40X). (b) Lung parenchyma with dilated capillaries and nonrefractile vacuole-like structures (20X).

A wedge biopsy tissue of the right lower lobe revealed intra-alveolar hemorrhage with dilated capillaries containing nonrefractile vacuole-like structures suggestive of deposition of lipoid material (Figures 2(a) and 2(b)). The lining of the dilated capillaries was positive for CD31 immunostain. Lymphatic spaces were also positive for D2-40 immunostain. No venoocclusive disease or features of arterial pulmonary hypertension were seen on EVG stain. These pathological findings raised concern for silicone embolism.

After discussion of the histological findings with the primary team, the patient was reinterrogated and she stated that she underwent twelve cosmetic subcutaneous silicone injections in the buttocks in the past by unlicensed practitioners. Her most recent injection occurred two months prior to this admission.

Within two weeks, she reported symptomatic improvement after supportive treatment and was discharged. Then, the patient was lost to follow-up.

### 2.2. Clinical Case #2

A 33-year-old HIV-positive transgender male-to-female patient presented with an 8-month history of shortness of breath, dyspnea, and productive green sputum.

On physical examination, the patient's vital signs were within normal limits. She was afebrile and had 97% of O_2_ saturation. The patient was not in acute distress; pulmonary breath sounds were clear to auscultation bilaterally without the presence of wheezing, crackles, or rhonchi. She had a regular heart rate and rhythm with no murmurs, rubs, or gallops. No signs of edema, clubbing, or cyanosis in her extremities were observed. She had good peripheral pulses and no gross neurological deficits.

There was high suspicion for silicone infiltration within the lung due to her recent history of cosmetic injections. Nonetheless, she was prescribed sulfamethoxazole and trimethoprim by her primary care physician (PCP), and the patient reported improving respiratory symptoms by being able to walk more than a mile, which was similar to her baseline. She denied any fevers or chills but reported weight loss and continued to indicate a cough productive of green sputum.

Chest X-ray showed bilateral hilar lymphadenopathy with increased interstitial markings. The chest CT without contrast showed mediastinal and hilar lymphadenopathies as well as ill-defined ground-glass opacities and nodular density, mainly along the pleural fissure and linear opacities on both lungs, predominantly in the right lower lung.

**Figure 3:**
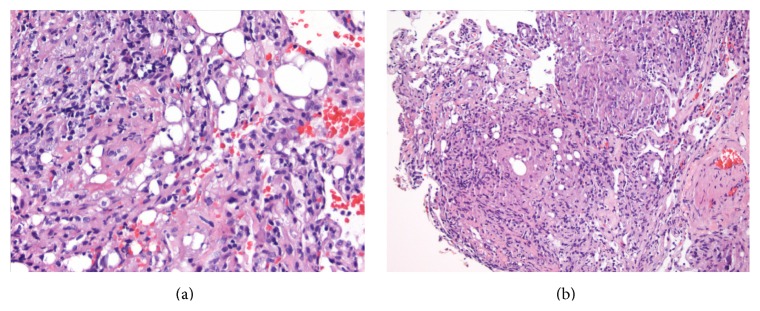
(a) Lung parenchyma with multiple vacuoles and dilated capillaries (40X). (b) Lung parenchyma with dilated capillaries and nonrefractile vacuole-like structures and histiocytic reaction (40X).

Laboratory tests conducted revealed WBC 3.4 × 10^9^ per liter, hemoglobin 14 mg/dL, and platelets 325,000. Sodium was 139 mmol/L, BUN 16, and creatinine 1.5 mg/dL. Liver function tests showed an AST of 73 U/L, ALT of 62 U/L, and an alkaline phosphatase of 390 U/L. Recent CD4 was decreased from 584 to 367. A bronchoscopy was performed. The microbiology cultures of the bronchoalveolar lavage fluid were negative, while the cytology sample revealed granulomas. The biopsy tissue showed granulomatous inflammation associated with silicone particles in the lung tissue (Figures 3(a) and 3(b)).

She was treated with supportive therapy due to the likely etiology of silicone embolism and the symptomatic improvement.

## 3. Materials and Methods

A PubMed search using the key words “silicone”, “embolism”, and “subcutaneous injection” for the period from January 2004 through May 2015 was performed. There were 16 articles describing 29 patients who had been hospitalized with respiratory syndromes after subcutaneous silicone injections. Each article was reviewed for demographic and clinical information to draw further understanding of modern presentations of silicone embolism. The resulting information was then compared with a previous review article written by Schmid et al., in which 33 patients presented with respiratory symptoms in the period from 1965 to January 2004 [2].

## 4. Results

**Figure 4:**
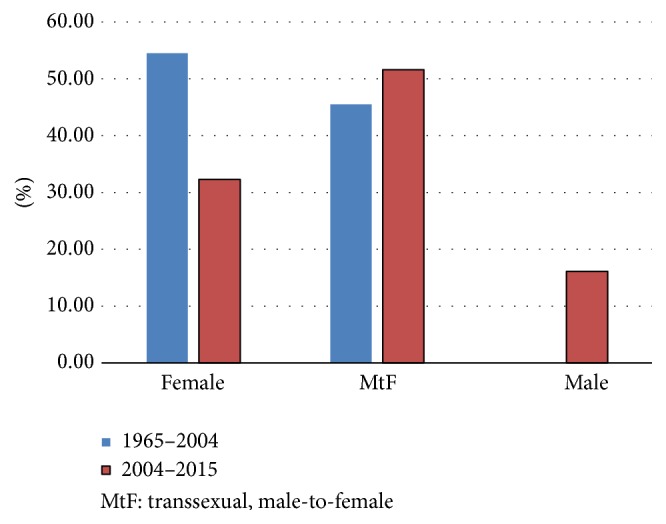
Difference in patient's demographic analysis by gender between two time periods (1965–2004 versus 2004–2015).

After comparison with the previous review article, we concluded that, over the last 12 years, the number of published articles per year describing silicone embolism has increased by almost threefold from that reported in the preceding 40 years. In our systematic review, including our two case reports, the literature has collectively described 16 transgender male-to-female patients (51.6%), 10 women (32.3%), and 5 men (16.1%) [2]. Among the cases in which the age was stated, the mean age was 31.2 years (age range, 22 to 53 years), which is about the same average age described by Schmid et al. [2]. However, there was an increase in proportion of transgender male-to-female patients from 45% to 51.6%, a decrease in female patients from 55% to 32.3%, and 5 cases of male patients (16.1%) (Figure 4). Within the male patients, additional social identifiers demonstrated that three were homosexual males and two were competitive bodybuilders who injected liquid silicone into their arms and pectoralis muscles.

**Figure 5:**
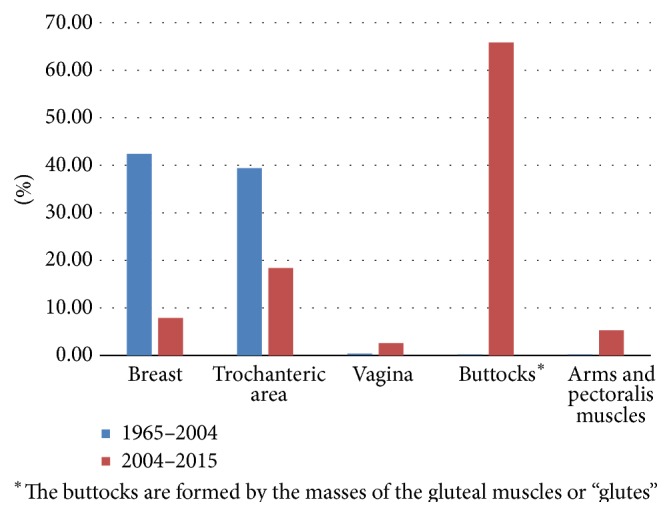
Difference in site of injections between two time periods (1965–2004 versus 2004–2015).

Another distinctive change over the last twelve years has been regarding the sites of injection. Previously, the majority of silicone injections were into the breast (14 patients or 45%) and trochanteric area (13 patients or 39%) while the buttocks (1 patient or 3%) were an uncommon site of injection. Presently, the buttocks (25 patients or 65.8%) are the most common site of injection, followed by the trochanteric (7 patients or 18.4%) and breast area (3 patients or 7.9%) (Figure 5). These results confirm a significant growth in the number of patients presenting with silicone embolism and the differences in the social context in which these patients may be present.

## 5. Discussion

Clinical findings associated with the diagnosis of silicone embolism include hypoxia (92%), dyspnea (88%), fever (70%), alveolar hemorrhage (64%), and cough (52%), all of which are similar to the presentation of an adipose tissue embolism [2–18]. As discussed by Schmid et al. [2], the pathophysiology of silicone embolisms is similar to that of fat emboli. Release of emboli leads to occlusion of the microvasculature, triggering an inflammatory response [19, 20]. Resulting hemorrhage and edema may lead to pulmonary consequences similar to that of respiratory failure and acute respiratory distress syndrome (ARDS) [21, 22]. Generally, silicone embolisms secondary to cosmetic procedures present in the first two days following a silicone injection. However, in some circumstances, such as those in our cases, the presentation may follow months after the injection [12, 23].

Computed tomography most often shows peripherally distributed ground-glass opacities associated with interlobular septal thickening, similar to what can be observed in some eosinophilic lung diseases and fat embolisms [8], as illustrated in our case (Figure 1(b)). However, a clinical and radiographic finding alone cannot provide a definitive diagnosis.

The characteristic histological findings for possible silicone embolism include dilated capillaries and nonrefractile vacuole-like structures. However, if pertinent information regarding prior cosmetic injections is not included in the clinical history, then further workup might prove necessary. Dark-field microscopy is helpful in revealing the refractile character of silicone [24, 25]. Using 48 to 72 hours modified Oil Red O stain in formalin-fixed, paraffin-embedded tissue sections may also be useful in revealing the presence of silicone embolus [24]. Positive results will aid in making a diagnosis, but negative results demand further evaluation.

The current literature describes the use of scanning electron microscopy with energy dispersive X-ray analysis and infrared spectroscopy, which will positively identify the sample as a type of silicone [16, 24]. Unfortunately, these procedures can only be performed by specially trained personnel and are typically only available at large medical centers. Bronchoalveolar lavage can be helpful when macrophages with intracytoplasmic inclusions of the substance are visible [23, 26]. However, the presence of intra-alveolar hemorrhage may obscure the cells and increase the difficulty of identifying the inclusions.

In our first case, physicians were uninformed of the patient's previous cosmetic silicone injections and the presentation was not a typical picture of pulmonary embolism. Therefore, pathological findings played a major role in determining an accurate diagnosis. Other entities may mimic silicone embolism, such as exogenous lipid pneumonia, bone marrow embolism, pseudo-lipid pneumonia, and bubble artefacts. Bubble artefacts are a result of escaping air from alveolar spaces during manipulation. Histologically, these appear as sharply demarcated spaces within an alveolar space to join an adjacent one or an alveolar sac. The spaces have no cellular lining and do not elicit histiocytic reaction (Figure 3(b)).

A review of the data illustrates the recent increase of complications after cosmetic silicone injection procedures, thus demonstrating the need for safer, standardized procedures and for reducing the number of unlicensed individuals performing them. This review highlights the importance of physicians to include silicone embolism in the differential diagnosis for patients with the discussed clinical presentation regardless of cosmetic surgical history.
